# Screening of cellulose-degrading yeast and evaluation of its potential for degradation of coconut oil cake

**DOI:** 10.3389/fmicb.2022.996930

**Published:** 2022-10-06

**Authors:** Zi-huan Fu, Jing Liu, Long-bin Zhong, Huan Huang, Peng Zhu, Cai-xing Wang, Xin-peng Bai

**Affiliations:** Engineering Research Center of Utilization of Tropical Polysaccharide Resources, Ministry of Education, Hainan University, Haikou, China

**Keywords:** coconut oil cake, crystallinity, cellulose-degrading bacteria, *Meyerozyma*, molecular sequencing, agro-industrial waste

## Abstract

Coconut oil cake (COC), a byproduct of oil extraction, contains high levels of cellulose. The aim of this study was to isolate a cellulose-degrading yeast from rotten dahlia that can effectively use COC as the only carbon source for cellulase secretion. Based on screening, *Meyerozyma guillermondii* CBS 2030 (*M. guillermondii*) was identified as a potential candidate, with the highest cellulolytic activity among the yeast strains isolated, with the carboxymethyl cellulase (CMCase) activity reaching 102.96 U/mL on day 5. The cellulose in COC samples was evaluated before and after degradation by *M. guillermondii*. Analysis based on field emission scanning electron microscopy (FESEM) revealed that the COC structure was changed significantly during the treatment, indicating effective hydrolysis. Fourier transform infrared spectroscopy (FTIR) of the modified functional groups indicated successful depolymerization of coconut cake. X-ray diffraction (XRD) and analysis of color differences established effective degradation of COC by *M. guillermondii*. The results demonstrate that *M. guillermondii* effectively secretes CMCase and degrades cellulose, which has important practical significance in COC degradation.

## Introduction

Coconut oil cake (COC), a byproduct of oil extraction, is a large agricultural and industrial waste. India and the Philippines are the primary producers of COC, manufacturing nearly 1,931,000 tons and 230,000 tons, respectively, after discharging the oil mechanically (Khot et al., 2015). Similarly, Hainan Province is the only tropical province in China that accounts for more than 90% of the country’s total coconut output, which is accompanied by a large amount of COC waste during coconut oil processing and manufacture. In the past, COC was used as an ingredient of low-cost animal feed because it contained a considerable amount of protein (20–25%) (Wong et al., 2020). Due to the recent tightening of aflatoxin B1 regulations, low levels of essential amino acids and poor palatability, the conventional application of COC in animal feed formulas is decreasing year by year, worldwide, underscoring the need for effective treatment of COC solid waste. In rural areas of China, the solid waste represented by COC is difficult to treat. The complex composition and the presence of rich polysaccharide fibers such as cellulose, which are difficult to degrade, lead to waste accumulation and environmental pollution (Zhang and Dong, 2022).

Cellulose is difficult to degrade due to its compact structure. Various methods, mainly physical, chemical and biological treatments, have been developed for cellulose degradation. Current studies are mainly focused on the treatment of solid waste *via* gas/steam explosion, which uses high temperature and pressure to soften lignocellulose, breaking the chemical bonds between cellulose crystals and cellulose, resulting in the cleavage of hemicellulose carboxyl groups (Tanpichai et al., 2019). However, harmful byproducts, such as acetic acid, furfural and hydroxymethylfurfural are produced during the process. The chemical method, based on acid or base treatments to break the glycosidic bonds, reduces the degree of cellulose polymerization and thus promotes degradation. Nevertheless, concentrated acids or bases are highly oxidative and corrosive, and are difficult to neutralize in the subsequent reactions, which leads to damage of the reaction vessels. Physical and chemical methods are effective; however, their high cost and the associated environmental issues are unsustainable (Dar et al., 2022).

Approximately 70% of organic waste is biodegradable. Biological methods include microbial fermentation and enzymatic hydrolysis utilizing microorganisms and their carbohydrate-active enzymes to depolymerize the lignocellulosic polysaccharides (Suchova et al., 2022). Compared with other methods, the biological method is more economical, safer and more environmentally friendly, and feasible under mild conditions (Danso et al., 2022). The biological method relies on identifying the appropriate microorganisms with efficient carbohydrate-active enzymes, capable of degrading cellulose and hemicellulose. Cellulase, the core enzyme for depolymerizing lignocellulosic polysaccharides, breaks down the β-1,4-bonds in cellulose polymers to release glucose units (de Paula et al., 2019). Cellulases are key biocatalysts in the degradation of lignocellulosic biomass for industrial applications, beginning with the animal feed industry in the early 1980s to the biorefining and biofuel industries today (Dadwal et al., 2020; Dar et al., 2022). The conversion of lignocellulose to biofuels through cellulases to address fossil fuel depletion and greenhouse gas emissions is currently a global concern (Melendez et al., 2022; Sohail et al., 2022). Cellulases are key biocatalysts in the degradation of lignocellulosic biomass for industrial applications, beginning with the animal feed industry in the early 1980s to the biorefining and biofuel industries today (Dadwal et al., 2020; Dar et al., 2022). There is a growing demand for cost-effective cellulases in the biofuel industry; however, the production process of cellulases is very expensive, accounting for 50% of the total hydrolysis costs. Therefore, it is a bottleneck limiting their industrial utilization requiring the manipulation of microorganisms to increase enzyme production (Sulyman et al., 2020; Bhardwaj et al., 2021). In recent years, many studies have focused on finding new microorganisms encoding cellulase genes and process optimization to improve the economics of cellulase production (Lodha et al., 2020; Bhardwaj et al., 2021; Sohail et al., 2022). Some studies applied genetic engineering to produce cellulase with high biomass saccharification at a low cost. Because microorganisms are the primary source of cellulolytic enzymes, exploring different ecological environments to find new candidate microorganisms will undoubtedly provide a basis for the development of more efficient enzyme systems and cellulase resources utilization (Prasad et al., 2019; Lodha et al., 2020; Suchova et al., 2022). In general, cellulose-degrading bacteria are mainly isolated from decayed leaves, plants and feces of herbivorous animals. Currently, researchers have excluded many capable microorganisms, including mostly bacteria and filamentous fungi (Suchova et al., 2022). Bacteria are often regarded as potential candidates for cellulose degradation because of their rapid reproduction and ability for recombination (Danso et al., 2022). The majority of cellulase-producing microorganisms are filamentous fungi, especially white rot fungi, which produce lignin-degrading enzymes except laccase (Tii et al., 2021). However, most of the bacteria have limited enzyme-producing ability, whereas filamentous fungi often require prolonged culture conditions up to 4–8 weeks to digest the substrates (Garcia-Torreiro et al., 2016). These factors significantly limit their efficiency and large-scale production. By contrast, the potential of yeast in cellulose degradation has yet to be fully understood or exploited. According to Suchova et al. (2022), the enzyme levels in polysaccharide-degrading yeast are less than the levels in filamentous fungi or bacteria. No more than 3,000 yeast species have been identified to date, with more than 90% of the existing fungal diversity requiring further development (Boekhout et al., 2022). Thus, newer yeasts with the ability to degrade polysaccharides and their overall biotechnological potential have yet to be established (Suchova et al., 2022).

Therefore, this study sought to identify a yeast from rotten plants with rapid enzyme production, high peak enzyme activity and highly efficient cellulose degradation in COC. The cellulose-degrading properties of the yeast isolated were investigated on COC before and after degradation using field emission scanning electron microscopy (FESEM), Fourier transform infrared spectroscopy (FTIR), X-ray diffraction (XRD) and analysis of color differences. The application of yeast, especially wild-type yeast, was intended to provide a technical solution for the management of solid waste with high cellulose content represented by COC, alleviate environmental pollution, and lay a foundation for possible practical applications in the future.

## Materials and methods

### Materials and chemicals

Coconuts were collected from the coconut processing plant in Wenchang, Hainan, China, and dried at 80°C for 8 h in a SHELLAB 1,445 oven provided by Zhaoxin Enterprise Co., Ltd. (Tianjin, China). After cold-pressed oil was obtained by YJY-Z260 screw press (Xiaogan, Hubei Province Yijiayi Machinery Equipment Group Co., Ltd.), the COC was immediately sampled. The FW177 Chinese herbal medicine crusher (Tianjin Meist Instrument Co., Ltd., China) was used to pulverize the coconuts into coarse powder until more than 90% of the material passed through a 40-mesh sieve to obtain a fine powder of skim COC. The rotten dahlias were obtained from the Hainan Botanical Garden in China and were immediately transferred to the laboratory for inoculation. The yeast genome was extracted using the magnetic bead method developed by Nano Magnetic Biotechnology Co., Ltd. (Wuhan, Hubei Province). Sodium carboxylmethylcellulose (CMC-Na) medium, Bengal red medium and Yeast extract peptone dextrose medium were obtained from Hydawson Technology Co., Ltd. (Haikou, Hainan province, China). Ethanol and DNS reagents were provided by McLean Biochemical Co., Ltd. (Shanghai, China). All reagents used were analytical grade with a purity of 95–98%.

### Screening of cellulolytic bacteria

#### Qualitative screening

**FIGURE 1:**
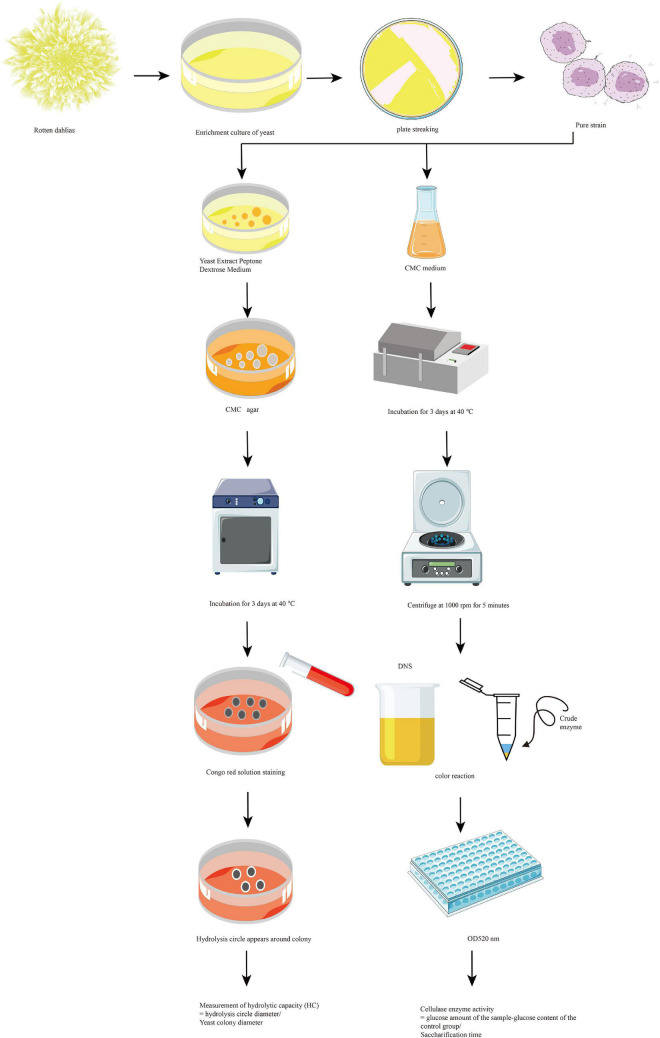
Qualitative and quantitative screening of cellulose-degrading bacteria.

The screening of cellulose-degrading bacteria is illustrated in Figure 1. The rotten dahlia sample was weighed and treated with sterile water. The mixture was fully shaken on a constant-temperature shaking table. The suspension was aspirated with a sterile pipette to obtain sample solutions with different dilutions and coat on Bengal red culture medium for 48 h. Single bacteria with yeast colony characteristics and large colonies were selected and repeatedly marked and separated on a yeast extract peptone dextrose culture medium until a single colony was obtained. Microscopic examination was used to establish pure bacterial cultures, which were preserved in Yeast extract peptone dextrose agar. The purified strains’ ability to secrete cellulase was assessed using the Congo red method (Danso et al., 2022). Pure isolates of single colonies were collected with sterile needles and inoculated separately on agar plates containing carboxymethylcellulose as the sole carbon source to screen the cellulolytic activity of the isolated yeasts. The inoculated plates were incubated at 37°C for 3 days. The yeast growth was observed until the strains that could grow stably on the medium were selected. The plates were stained with a 10 mL aliquot of Congo red dye solution (2.5 g/L) for 30 min, the dye solution was discarded, and the cultures were washed with 15 mL of 1 M NaCl for 10 min. The cellulose degradation activity of yeast was analyzed by measuring the CMC clearance zone around the yeast colony vs. the yeast colony diameter in millimeters. In addition, the hydrolytic capacity (HC) was estimated by the ratio of CMC clearance zone to yeast colony diameter (Danso et al., 2022). The potential yeast with the highest activity (highest HC value) among the isolated strains was selected based on plate assays, and further analyzed and characterized.

#### Quantitative screening (determination of carboxymethyl cellulase)

In this study, the method of Li et al. (2016) was used to determine the ability of the selected strains to secrete carboxymethyl cellulase (CMCase). CMC-Na was dissolved in sodium citrate buffer (50 mM, pH 5.5), containing 1.5 mL of 0.5% CMC-Na solution mixed with 0.5 mL crude enzyme solution of the test strain. The mixture was incubated in a water bath at 50°C for 30 min for enzymolysis. The reaction was terminated with 1.5 mL of DNS reagent, and the mixture was incubated at 100°C for 10 min. The reducing sugar released was measured with a UV-Vis spectrophotometer (Synergy LX, Blotek, USA) at 520 nm. The unit enzyme activity was defined as the amount of enzyme required to release 1 μmol of reducing sugar (calculated as glucose) per minute during the reaction. The strain with the highest enzyme activity of CMCase was selected for the next study.

### Fermentation enzyme production

The enzyme production by the selected strains incubated with the COC powder was tested. The strains were cultured at 40°C. The enzyme production by the strains was monitored every 24 h from day 3 of culture. The fermentation supernatant was used as the enzyme solution for determination of enzyme activity using the method described in section “Screening of cellulolytic bacteria.”

### Molecular screening of strains

The DNA of the selected yeast strains was extracted using the magnetic bead method described in the genome extraction kit of the product manual. Based on the conserved sequences of ITS1 and ITS4, the primers were designed, including the forward primer ITS1: 5’-TCCGTAG?GTGAACCTGCGG-3’and the reverse primer ITS4: 5′-TCCTCC?GCTTATTGATATGC-3′. The yeast DNA was amplified *via* polymerase chain reaction (PCR), and the purified PCR products were sent to Lifeite Biotechnology Co., Ltd. (Hainan, China) for DNA sequencing. The sequencing results were compared in GenBank^1^ through BLAST database, and the phylogenetic tree was constructed using MEGA × software (version 5.0, Mega Limited, Auckland, New Zealand). Neighbor-Joining (NJ) method was used to build trees, and the bootstrap was set to 1,000 (Kumar et al., 2018).

### Characterization of the coconut oil cake before and after degradation

#### Field emission scanning electron microscopy

FESEM (Verios G4 UC, Thermo Scientific, USA) was used to investigate changes in surface morphology in the untreated and treated COC samples. COC samples were mixed with 2.5% glutaraldehyde solution at 4°C for 24 h. The mixtures in turn were treated with 50, 70, 80, 90, and 95% ethanol solutions (v/v) for l5 min, followed by anhydrous ethanol for 20 min (Li et al., 2022). Samples were stored at –40°C for 4 h and dehydrated in a vacuum freeze-dryer. Samples were wrapped with carbon black tape and coated with gold-palladium. The surface morphology of the samples was analyzed using FESEM.

#### Fourier transform infrared spectroscopy

The changes in the functional groups of the COC samples before and after hydrolysis were analyzed with FTIR (Nicolet iS50, Thermo Fisher Scientific, USA). The untreated and treated COC powder samples were treated with potassium bromide (KBr) particles for the FTIR analysis (Dar et al., 2022). Briefly, 6 mg of dried COC sample and 600 mg of KBr were fully ground in an agate mortar and pressed under 200 kg cm^–2^ for 2 min for tableting. Tablets were transferred into the sample tray of the FTIR, under the following settings: resolution 4 cm^–1^, scan 32, and scan range 400–4,000 cm^–1^. The sample tray without any sample was used to obtain the background spectrum.

#### X-ray diffraction analysis

The crystallinity of the COC samples before and after MH10 fermentation was measured *via* XRD (Smart Lab, Rigaku, USA) under the following settings: voltage 40 kV, current 40 mA, scan range 5 ∼ 40°, and scan step 0.02°. The sample crystallinity was calculated according to the Segal equation (Ma et al., 2022):


(1) $$\text{CrI}=\frac{I_{002}-I_{a\,m}}{I_{002}}\times 100$$

In the equation above, CrI is the relative crystallinity of the sample; *I*_002_ denotes the diffraction peak intensity of the crystalline region, with a diffraction angle of about 2θ = 22°; *I*_*am*_ represents the diffraction peak intensity of amorphous region, with a diffraction angle of about 2θ = 18°.

#### Color

The color of COC powder before and after degradation was measured using a chroma meter (CR-10, Konica Minolta, Japan). The chroma meter was calibrated with a white tile before use, and the raw COC powder was used as the negative control. The CIELAB color system L* (lightness-darkness), a* (redness-greenness) and b* (yellowness-blueness) values were recorded 9 times.

### Statistical analysis

All experiments were repeated at least three times, and the average value was used. The correlation was analyzed using GraphPad Prism software (version 8.0.2, GraphPad Software, USA). A significant value was defined as a *P*-value < 0.05. Differences between means of cellulose activity were performed *via* unpaired *t*-test at *p* < 0.05, suggesting statistical significance.

## Results and discussion

### Qualitative and quantitative screening

Lignocellulose biomass (LCB) is mainly composed of three polymers: cellulose (40–60% by dry weight), hemicellulose (20–35% by dry weight), and aromatic polymer lignin (15–40% by dry weight) (Zoghlami and Paes, 2019). LCB is the most abundant raw material on the earth, with an annual output of about 200 billion tons. It is often composed of various plant residues, most of which are agricultural wastes, accumulating in the form of LCB every year (Sharma et al., 2022). Among them, cellulose is directly converted to resources for degradation, saccharification and other processing. However, cellulose is difficult to hydrolyze due to its structural characteristics. In order to address this limitation, organisms are used to separate cellulose components, produce cellulolytic enzymes, and hydrolyze cellulose-rich waste materials to release glucose. The decomposition of yeast using natural cellulose yielded only a few products of cellulose degradation. Therefore, many studies report that yeast cannot secrete cellulose (Sohail et al., 2022). Most of the yeasts diverged from the common ancestor and lost the ability to degrade cellulose. However, this ability is conserved in many filamentous fungi, although the list of cellulose-degrading yeasts has not grown substantially in the past few years (Suchova et al., 2022).

**FIGURE 2:**
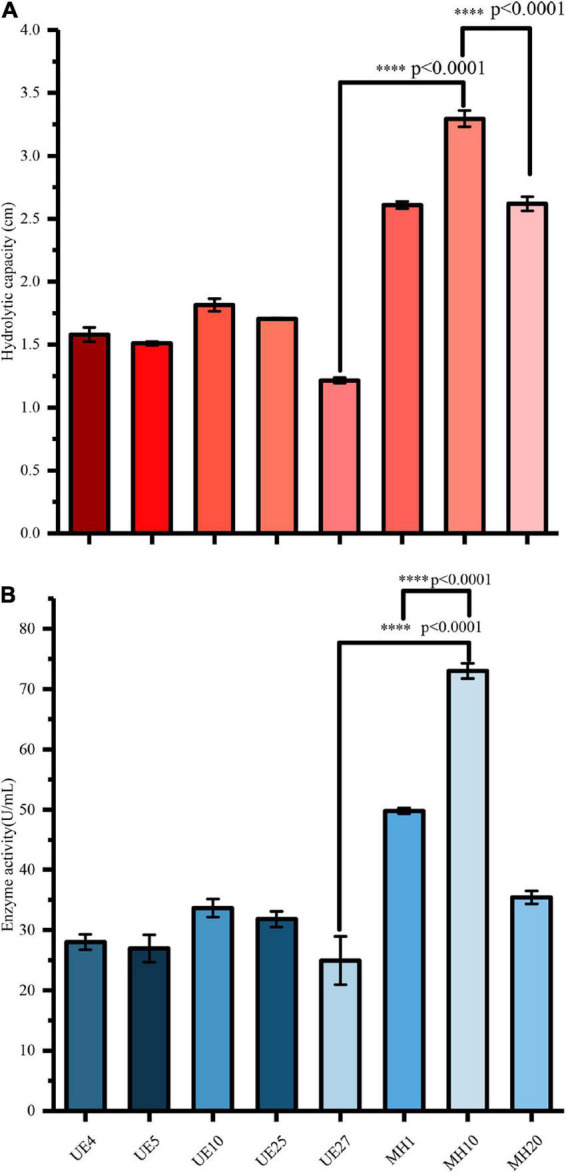
Comparison of enzyme production capacity of different strains exhibiting cellulase hydrolysis **(A)** and cellulase activity **(B)**. ^∗∗∗∗^*p*-value < 0.0001.

Accordingly, sodium carboxymethyl cellulose (CMC) was used as the sole carbon source during the initial experimental screening. Thirty yeasts were isolated from halophytes, and their growth on the plate was monitored. Different colonies were selected and purified by streaking on various agar media. Among the 30 strains, 8 strains, which expressed cellulase activity during the qualitative analysis (Congo red test), were selected. After culturing to determine humidity at constant temperature for 3 days, the 8 strains produced obvious degradation circles after Congo red staining, showing strong cellulose hydrolysis ability. As shown in Figure 2A, the capacities of the strains UE4, UE5, UE10, UE25, UE27, MH1, MH10, and MH20 for cellulose hydrolysis were 1.58, 1.51, 1.815, 1.705, 1.215, 2.61, 3.295, and 2.62 cm, respectively. Among the eight strains which produced transparent circles, MH10 showed higher hydrolytic ability, suggesting higher cellulase activity. However, this cannot fully represent the enzyme-producing ability of the strain, because the size of hydrolysis circle around the colony is not only related to the enzyme activity, but also to the strain characteristics. Therefore, further quantitative screening of the enzyme activity by spectrophotometry was needed. As shown in Figure 2B, the CMCase activities of UE4, UE5, UE10, UE25, UE27, MH1, MH10, and MH20 were 28.01, 26.95, 33.64, 31.8, 24.95, 49.78, 72.98, and 35.42 U/mL, respectively.

Our data also showed that the cellulase hydrolytic abilities of MH10 were 108.54, 118.21, 81.54, 93.26, 171.19, 26.24, and 25.76% higher than those of UE4, UE5, UE10, UE25, UE27, MH1, and MH20, respectively. The enzyme activities of MH10 were 160.5, 170.8, 116.94, 129.53, 46.6, and 106.04% higher than those of UE4, UE5, UE10, UE25, UE27, MH1, and MH20, respectively. In addition, statistical analysis showed that the cellulase hydrolytic ability and cellulase activity of MH10 was significantly higher than that of the other strains (*P* < 0.001) (Figure 2B). Following culture on CMC-Na agar medium, the MH10 strain exhibited the highest cellulase hydrolysis in qualitative (Congo red test) and quantitative tests. Therefore, the most potent MH10 strain was selected for identification and further studies. Accordingly, based on the qualitative analysis of Congo red, Danso et al. (2022) selected a bacterium with the highest cellulase yield for degradation and saccharification of wheat straw, to release reducing sugar, and further conversion to bioethanol. In the study of yeast degrading polysaccharides, *Saccharomyces cerevisiae* was preferred because of its high-efficiency ethanol production and strong enzyme-producing characteristics (Suchova et al., 2022). Using yeast strains isolated from mushroom farms, Amadi et al. (2020) produced cellulase, xylanase, and ligninase from lignocellulose waste and the yeast was finally identified as *Saccharomyces cerevisiae* SCPW 17.

### Determination of fermentation enzyme production capacity

**FIGURE 3:**
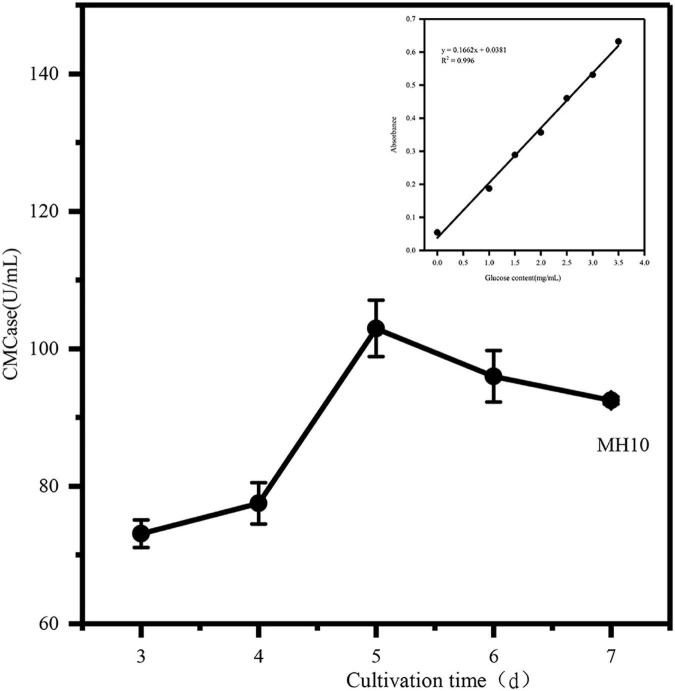
Determination of CMCase enzyme activity in COC fermentation.

Every year, tons of agricultural wastes, such as coconut dregs, are produced worldwide. Thus, there is an urgent need for efficient and low-cost cellulolytic bacteria to degrade solid wastes. Biological treatments are needed to address this challenge. COC is rich in cellulose, suggesting the need for cellulase synthesis for efficient degradation of COC. Strain degradation is a continuous process with several limitations for use in instantaneous enzyme activities during initial screening of the strain. It is a reliable theoretical approach for evaluating cellulose degradation ability of the strain by continuously measuring its enzyme activity. Therefore, in this study, the crude cellulase (cellulase), which was used to release the reducing sugar in COC, was produced by screening and analyzing the cellulose-producing strain qualitatively and quantitatively during the early stage, in order to determine whether the strain meets the actual production demand and has the ability to efficiently degrade the cellulose. The enzyme production curve of the strain was determined, and the production efficiency was evaluated. As can be seen from the Figure 3, MH10 produces relatively stable cellulase with high peaks of cellulase activity, reaching about 102.96 U/mL on day 5, decreasing to 95.9 U/mL on day 6, followed by stable cellulase production. This result might be attributed to nutrient consumption in the culture medium and the enormous growth of the yeast, resulting in excessive bacterial concentrations and a decrease in CMCase activity. The growth trend was obvious in the first 5 days, and the potential cellulose degradation by the strain was established by the short-term and high-yield enzyme activity. The changing trend of CMCase activity reported by Ali et al. (2019) is similar to that of this study. In the first 10 days of culture, the bacteria used cellulose as a carbon source for growth and metabolism, suggesting high cellulose degradation activity.

### Molecular biology of strains

**FIGURE 4:**
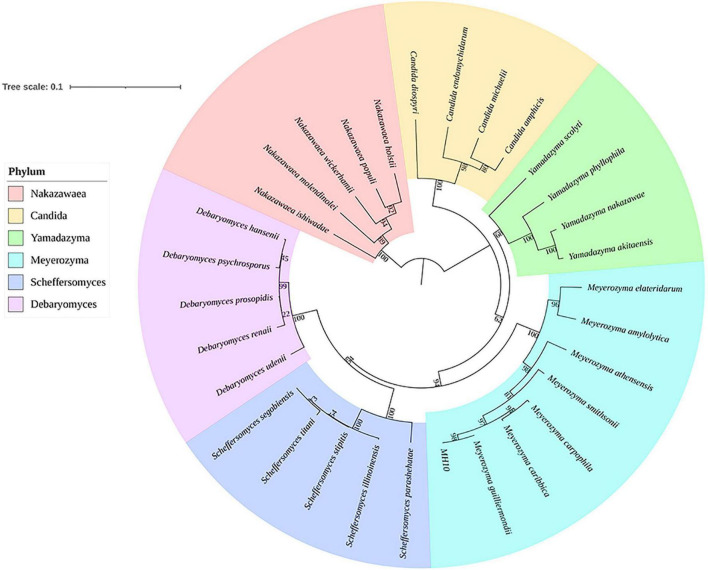
Phylogenetic affiliation of MH10, based on the phylogenetic tree generated using Mega X software.

During the qualitative and quantitative analysis of enzyme, the strain MH10 showed the highest cellulase yield. Based on these attributes, MH10 was selected for authentication. The 18S rDNA gene sequence of MH10 determined *via* NCBI BLAST search showed strong similarities (100%) with *Meyerozyma guilliermondii*. The phylogenetic tree constructed *via* NJ method (Figure 4) also showed that MH10 is located in the pedigree of *Meyerozyma*. Therefore, it can be concluded that MH10 is a species belonging to *Meyerozyma*. The close relationship between *Meyerozyma* and *Pichia* indicates similar genus. *Meyerozyma* was upgraded to an independent genus in 2010. Generally speaking, the species grouped under the genus *Meyerozyma* are widely distributed in nature. Knob et al. (2020) isolated *M. guilliermondii* from oil plant wastewater and produced efficient acidic lipase at low cost using abundant and highly polluting waste materials, which showed prospects for potential application in feed industry. Rehman et al. (2021) used *Meyerozyma* species isolated from a site in Pakistan contaminated with crude oil to investigate its ability for degradation of crude oil. The results showed that *Meyerozyma* species used crude oil to produce acidic and lactose from *Sophora japonica*. Based on the analyses of weight and GC-MS, *Meyerozyma* species exhibited a biodegradation efficiency of 87%, suggesting a strong potential for application in the bioremediation of hydrocarbon-contaminated sites.

In addition, we evaluated the cellulose degradation efficiency of the yeast in this study, comparing the cellulase activity of *M. guillermondii* with that of other yeast strains. As shown in Table 1, the potent strain in this study effectively hydrolyzed COC without pretreatment and recorded significant cellulase activity compared to other studies. In addition to the strains’ characteristics, coconut waste as a natural renewable raw material biogenic source of CMC was a factor that led to the maximum cellulase yield found (Pham et al., 2022). Dey et al. (2018) and Pham et al. (2022) hydrolyzed COC by bacteria and fungi, respectively, expressing 650 and 93 U/mL of CMCase activity, respectively. This study confirms that enzyme production from cheap and abundant coconut waste (COC) is possible using yeast, overcoming the use of cellulase in production hindered by the expensive cost of the substrate.

**TABLE 1:** Comparison between the largest cellulase produced by *M. guillermondii* CBS 2030 and early discovery.

Bacterial strain	Isolated from	Cellulase activity (U/mL)	Substrate	Pre-treatment	References
*Meyerozyma guillermondii* CBS 2030	Rotten dahlias	102.96	Coconut oil cake	NA	This study
*Candida tropicalis* MK-118	Soil	17.39	Sugarcane bagasse	NaOH	Shariq and Sohail, 2020
*Sporobolomyces poonsookiae* Y08RA07, *and Cryptococcus flavescens* Y08RA33	Soil	1.40 and 1.66	Bamboo leaf	NA	Sohail et al., 2022
*Trichosporon asahii*	Forest soil samples	35.70	Napier biomass	NA	Valliammai et al., 2021
*Cystobasidium oligophagum*	Soil rich in cellulosic waste	0.072	CMC	NA	Vyas and Chhabra, 2017
*Candida* sp. 05-7-186T, *Candida easanensis and Candida* sp. ST-390	Soil, tree barks, and insect frass	0.224, 0.238, and 0.226	CMC	NA	Thongekkaew and Kongsanthia, 2016
*Trichosporon laibachii* MG270406-1A14	Decaying leaves, wood, and ant nest	0.3	CMC	NA	Giese et al., 2017

NA, not available.

### Characterization of coconut oil cake before and after degradation

#### Field emission scanning electron microscopy analysis

**FIGURE 5:**
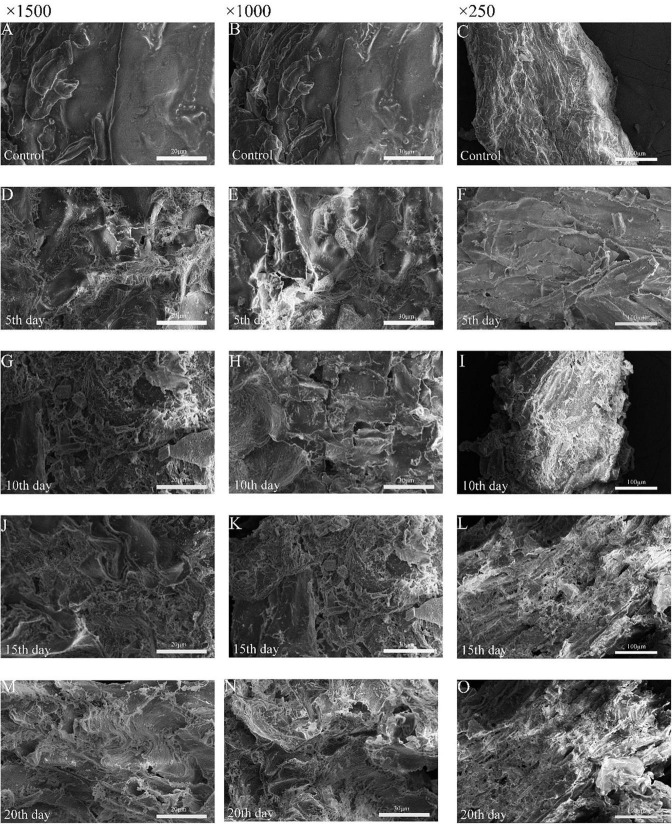
Field emission scanning electron micrographs before degradation **(A–C)**, degradation d 5 **(D–F)**, degradation d 10 **(G–I)** and degradation d 15 **(J–L)**, and degradation d 20 **(M–O)**.

COC samples were treated with *M. guillermondii via* biological liquid fermentation. Figure 5 shows the surface structure of COC scanned during *M. guillermondii* liquid fermentation at different time periods and untreated samples by FESEM, respectively. The COC samples were scanned with electron microscope every 5 days. Compared with the COC treated with *M. guilliermondii*, the morphological structure of untreated COC was regular and dense, and the surface was smooth and flat with better integrity (Mihajlovski et al., 2021). However, treatment with *M. guillermondii* for 5 days led to irregular rough structures with uneven cracks and pores on the surface of COC, possibly due to the degradation of COC and the change in density induced by the hydrolysis of *M. guillermondii* (Tsegaye et al., 2018). The trace degradation on day 10 of the liquid fermentation was deeper than on day 5, showing the characteristic honeycomb structure with a large number of holes and cracks (Figures 5G–I), and partial shedding of the outer layer. Treatment with *M. guilliermondii* weakened the protective layer on the surface of COC and shedding of the protein or starch around COC, facilitating the degradation of cellulose and hemicellulose in COC by cellulose- and hemicellulose-degrading enzymes (Zheng and Li, 2018). During the late stage of fermentation (days 15–20), the outer layer of fiber was fragmented, and led to the formation of multiple cracks and gullies. Thus, the FESEM analysis revealed a loosening of the cellulose structure, thereby facilitating binding with the active sites of cellulase. Fermentation and utilization of the strains disrupted the cellulose in the middle, and apparently altered the general structure and properties mainly because *M. guilliermondii* used cellulose for metabolism during cultivation, which led to the degradation of COC cellulose. The destruction of fibers demonstrated that the microbial attack altered the fibrils at the molecular level. The study of wheat straw degradation revealed similar findings (Danso et al., 2022) following treatment with *Streptomyces* (Baramee et al., 2020). Likewise, the channels and pores which were formed by the microorganisms attached to the substance exposed the internal fibers of cellulose (Dar et al., 2022) concluded that, which indicated that the enzymatic hydrolysis of biodegradable polymers was mainly mediated *via* surface erosion.

#### Fourier transform infrared spectroscopy

**FIGURE 6:**
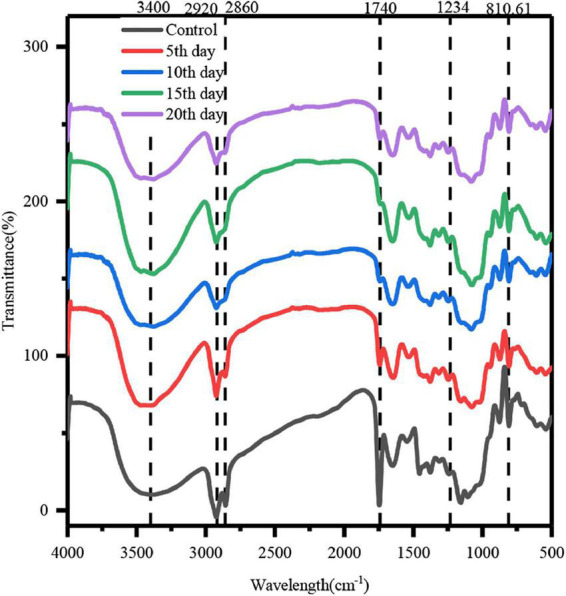
Infrared spectral analysis of COC before and after biological treatment.

In order to study the mechanism of COC degradation by MH10, the COC was analyzed with FTIR before and after strain treatment. The FTIR spectra of polysaccharides (cellulose and hemicellulose) and lignin showed typical peaks of cellulose functional groups, which were generally similar before and after degradation (Figure 6). However, a few characteristic bands showed altered absorbance or wave number, which confirmed the effects of MH10 on the COC structure. The peak at 810.61 cm^–1^ indicated a tensile vibration of β-glucosidic bond in the polysaccharide, the strength of which was gradually weakened with the duration of fermentation, suggesting that MH10 disrupted the hydrogen bond between cellulose and hemicellulose (Zheng and Li, 2018). The absorption peak at 1,740 cm^–1^ was characterized by hemicellulose acetyl C = O (Baramee et al., 2020). The intensity of this peak decreased significantly with extended fermentation, indicating that MH10 exhibited strong selectivity for the degradation of hemicellulose in COC. The degradation of a large number of hemicellulose components in COC led to partial depolymerization of acetyl functional groups in hemicellulose. After the fermentation, the absorption peak of COC treated with MH10 changed at 2,920 cm^–1^ compared with unfermented COC, which indicated that cellulose polysaccharides were depolymerized and degraded during fermentation. The wide absorption band at 3,550–3,200 cm^–1^, which showed the maximum strength at about 3,400 cm^–1^, is attributed to the O-H stretching of hydrogen bound to hydroxyl groups, mainly from cellulose and hemicellulose, and the narrow width following degradation was due to the enzymatic hydrolysis and degradation of cellulose (Zheng and Li, 2018).

The band at 2,860 cm^–1^ represents the C-H vibration of methylene group (Zhao et al., 2013). This peak was detected in the spectra of COC before and after fermentation, but its intensity during fermentation was lower, indicating destruction of a few hydrogen bonds between cellulose chains.

The C-O tensile band around 1,243 cm^–1^ represents hemicellulose and cellulose (Dar et al., 2021). In addition, the peak value was about 1,600 cm^–1^, which corresponded to the aromatic ring vibration of lignin, indicating the presence of lignin in the degraded solid (Lu et al., 2022). FT-IR analysis of modified functional groups showed successful depolymerization of COC. These findings demonstrate that *M. guilliermondii* effectively utilizes hemicellulose and cellulose components of COC, which is consistent with the results of previous CMCase enzymatic studies and FESEM. The foregoing observations strongly suggest that *M. guilliermondii* is capable of transforming industrial and agricultural waste-coconut cake into sugar and other commodities.

#### X-ray diffraction

**FIGURE 7:**
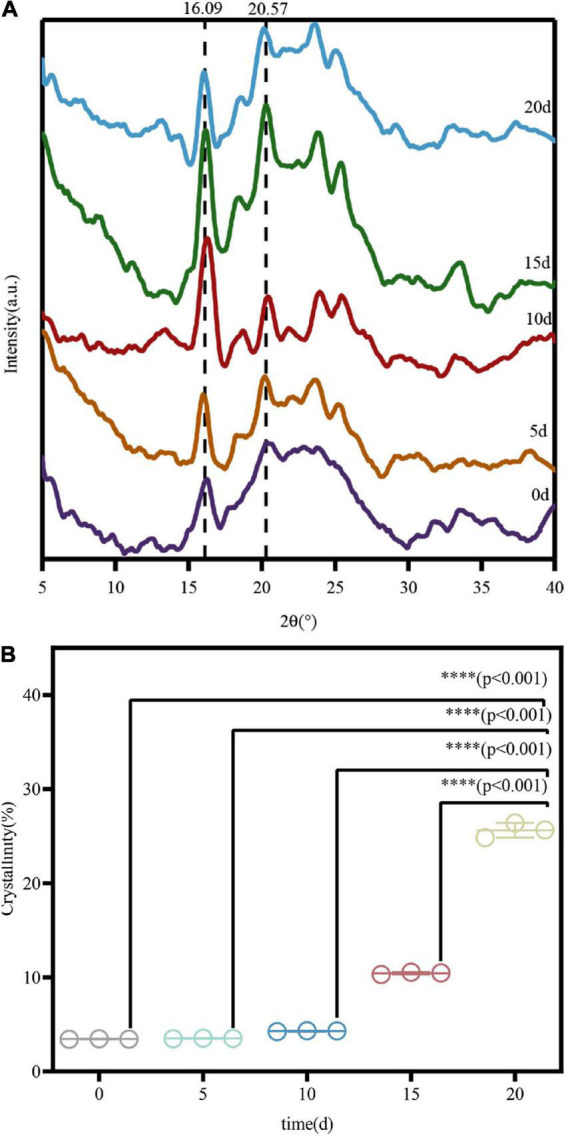
X-ray diffraction **(A)** and crystallinity **(B)** of COC at different treatment times. ^∗∗∗∗^*p*-value < 0.0001.

The crystallinity of cellulose is an important characteristic that affects cellulase hydrolysis in lignocellulosic materials. XRD was used to analyze the changes in the crystal structure of COC cellulose before and after degradation by *M. guilliermondii*. The results presented in Figure 7A show regular broad peaks at 14.8–28° in all samples, suggesting typical cellulose crystal form and strong peaks at about 20.57 and 16.09° (Zheng and Li, 2018). After 20 days of fermentation by *M. guillermondii*, the position of the characteristic diffraction peak was basically the same as that of the untreated COC, but with a difference in intensity, which indicated incomplete disruption of the crystal structure of cellulose during the fermentation. The CrI value was calculated according to the XRD diffraction pattern. The results shown in Figure 7B indicate that compared with unfermented COC, the CrI value increased from 3.44 to 27.2%. Based on the analysis of previous experiments, the selective degradation by *M. guillermondii* led to hydrolysis of cellulose and hemicellulose in the amorphous region and dissolution to soluble molecules and small sugar molecules, which increased the proportion of cellulose in the crystalline region, thus increasing the CrI of the sample. Cellulose obtained from the amorphous region is more vulnerable to microbial and enzymatic attack compared with those derived from the crystallization zone, which leads to increased surface porosity of the cell wall structure, resulting in large-scale degradation. Therefore, the rate of enzymatic hydrolysis is greatly affected by the crystallinity of cellulose molecules. In addition, pretreatment of the waste straws with sulfuric acid (Gu et al., 2021) increased the CrI significantly compared them with the control group without pretreatment. Thus, during pretreatment with the dilute acid, the degradation of amorphous biomass (including hemicellulose) exposed additional binding sites, which led to the increase of CrI, resulting in effective cellulase binding. Therefore, *M. guilliermondii* preferentially degrades cellulose and hemicellulose in the amorphous region. Our results are also consistent with those reported by Dar et al. (2018), suggesting a similar increase in the crystallinity index of sawdust after treatment with *Klebsiella* MD21.

#### Color differences

**FIGURE 8:**
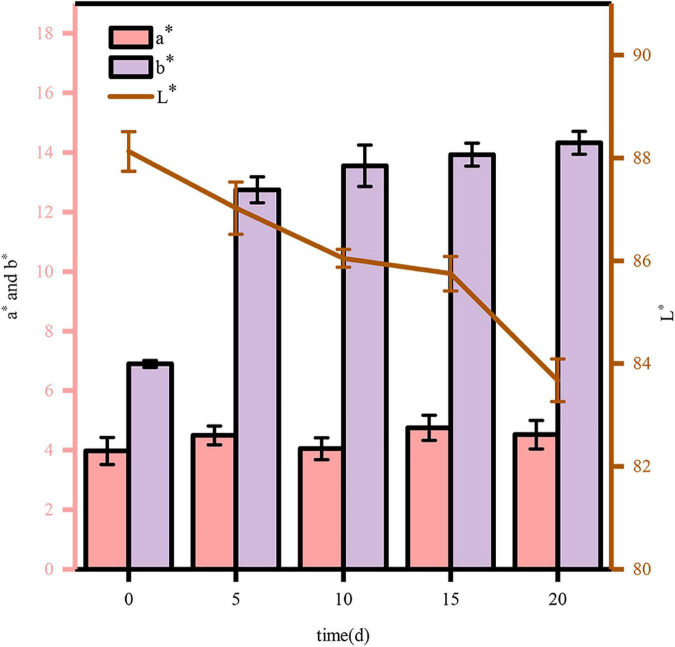
Changes in color during COC fermentation.

Analysis of color differences can be used to determine the degradation of COC with high accuracy and strong reproducibility, which reflects the degree of COC degradation. Therefore, during the degradation, the change in chromaticity should be measured in real time. According to the methods mentioned above, the chromaticity should be measured every 5 days. The results shown in Figure 8 demonstrate that the enzymatic hydrolysis before and after fermentation promote color changes in COC samples. When the value of L* increases, the sample turns white and bright, whereas a decreased L* value leads to black and dark colors. During the fermentation of COC, the L* value decreased gradually, indicating that the COC turned black and dark eventually during the degradation. The results showed browning and Maillard reactions during the cellulase hydrolysis (Perez-Jimenez et al., 2014). In contrast, the value of b* (representing blue or yellow) after degradation was larger than the levels before. These experimental results were consistent with those reported previously (Zheng and Li, 2018). An increase in the value of a* indicates conversion to red color, while a decrease indicates green. The value basically does not change during the fermentation (0∼20 days), indicating that the color barely changes at this time. The high efficiency of *M. guilliermondii* in COC was reconfirmed *via* colorimetry.

## Conclusion

In summary, in this study, a cellulase-producing yeast, which was identified as *M. guilliermondii* based on 18S rDNA, was successfully screened in the potential low-cost resource bank of rotten plants *via* qualitative and quantitative analysis. This strain can effectively use COC as the sole carbon source and secrete cellulase significantly. The degradation of COC by *M. guilliermondii* was further characterized by FESEM, FTIR, XRD and differential color analysis, and the inherent ability to decompose COC was revealed. This study explored the ability of *M. guilliermondii* to degrade cellulose for the first time, adding to the list of cellulose-degrading yeasts and confirming that isolation and screening of high cellulase producing yeasts from nature is an economically viable route. The discovery of this study opens up a new avenue for the search for cellulose-degrading yeasts, which is of great relevance for the degradation of agro-processing by-products.

## Data availability statement

The data presented in this study are deposited in the NCBI repository (https://www.ncbi.nlm.nih.gov/), accession number: OP445243.

## Author contributions

Z-HF: methodology and writing—original draft. JL: data curation and formal analysis. L-BZ: assistance with software and writing—review and editing. HH: funding acquisition and validation. PZ: assistance with software and data curation. C-XW: assistance with software. X-PB: funding acquisition, validation, and writing—review and editing. All authors contributed to the article and approved the submitted version.

## Footnotes

1
http://blast.ncbi.nlm.nih.gov/Blast.cgi
